# Better Long-Term Survival in Young Patients with Non-Metastatic Colorectal Cancer after Surgery, an Analysis of 69,835 Patients in SEER Database

**DOI:** 10.1371/journal.pone.0093756

**Published:** 2014-04-03

**Authors:** Qingguo Li, Guoxiang Cai, Dawei Li, Yuwei Wang, Changhua Zhuo, Sanjun Cai

**Affiliations:** 1 Department of Colorectal Surgery, Fudan University Shanghai Cancer Center, Shanghai, People’s Republic of China; 2 Department of Oncology, Shanghai Medical College, Fudan University, Shanghai, People’s Republic of China; University of California, Irvine, United States of America

## Abstract

**Objective:**

To compare the long-term survival of colorectal cancer (CRC) in young patients with elderly ones.

**Methods:**

Using Surveillance, Epidemiology, and End Results (SEER) population-based data, we identified 69,835 patients with non-metastatic colorectal cancer diagnosed between January 1, 1988 and December 31, 2003 treated with surgery. Patients were divided into young (40 years and under) and elderly groups (over 40 years of age). Five-year cancer specific survival data were obtained. Kaplan-Meier methods were adopted and multivariable Cox regression models were built for the analysis of long-term survival outcomes and risk factors.

**Results:**

Young patients showed significantly higher pathological grading (p<0.001), more cases of mucinous and signet-ring histological type (p<0.001), later AJCC stage (p<0.001), more lymph nodes (≥12 nodes) dissected (p<0.001) and higher metastatic lymph node ratio (p<0.001). The 5-year colorectal cancer specific survival rates were 78.6% in young group and 75.3% in elderly group, which had significant difference in both univariate and multivariate analysis (P<0.001). Further analysis showed this significant difference only existed in stage II and III patients.

**Conclusions:**

Compared with elderly patients, young patients with colorectal cancer treated with surgery appear to have unique characteristics and a higher cancer specific survival rate although they presented with higher proportions of unfavorable biological behavior as well as advanced stage disease.

## Introduction

Colorectal cancer (CRC) is one of the most common malignancies and is ranked as the third leading cause of cancer-related deaths in the USA [1]. The incidence of CRC in Asian countries is increasing rapidly and has been considered to be similar to that of the Western countries [2], [3]. Generally, CRC is thought to be a malignancy affecting mostly on the elderly persons, with more than 90% of patients being diagnosed after age 55 years [4]. The 2010 Annual Report to the Nation on Cancer celebrated a steady decline in the incidence of CRC in USA [5]. In sharp contrast to overall trends, the incidence of CRC in young patients appears to be increasing [5], [6], [7]. The incidence of the disease, considering patients aged between 20–40 years of age increased by 17% during the period between 1973 and 1999 [7].

CRC in the young generally regards as a higher prevalence of mucinous or poorly differentiated tumors including signet ring carcinoma and later stage, which tend to have a poorer prognosis compared to elderly patients [8], [9], [10], [11]. Some authors, however, argued that compared with the elderly patients, although the young ones have unfavorable cliniopathological characteristics, they have better, at least no worse, long-term survival rates than elderly [12], [13], [14]. Most of the published studies on CRC in young patients are single-institution experiences or limit sample sizes. In this regard, we used data from the Surveillance, Epidemiology and End Results (SEER) registries to analysis age role on CRC long time survival after surgery.

## Materials and Methods

### Patients

The current SEER database consists of 17 population-based cancer registries that represent approximately 26% of the population in the United States. The SEER data contain no identifiers and are publicly available for studies of cancer-based epidemiology and health policy. The National Cancer Institute’s SEER*Stat software (Surveillance Research Program, National Cancer Institute SEER*Stat software, www.seer.cancer.gov/seerstat)(Version 8.1.2) was used to identify patients whose pathological diagnosis as invasive CRC (C18.0–20.9) between 1988, and 2003. Only patients who underwent surgical treatment with age of diagnosis between 18 and 74 years were included. Patients were excluded if they had in situ or incomplete TNM staging, with distant metastasis (M1), no evaluation on lymph nodes (LNs) or differentiation grade or histological type pathologically, died within 30 days after surgery, or multiple primary malignant neoplasm as determined by Extent of Disease Codes. Age, sex, race, TNM stage, tumor grade, tumor location, CRC–specific survival (CCSS) was assessed. The lymph nodes ratio (LNR) was calculated as the number of positive regional nodes divided by the number of regional nodes examined and defined as the rN classification. Adjuvant chemotherapy was not evaluated as the SEER registry does not include this information. TNM classification was restaged according to the criteria described in the American Joint Committee on Cancer (AJCC) Cancer Staging Manual (7th edition, 2010). The primary endpoint of study is CCSS which was calculated from the date of diagnosis to the date of cancer specific death. Deaths attributed to the cancer of interest are treated as events and deaths from other causes are treated as censored observation.

This study was based on public data from the SEER database and we had got the permission to access the research data files with the reference number 12768-Nov2012. It didn’t include interaction with human subjects or use personal identifying information. The study did not require informed consent and was approved by the Review Board of Fudan University Shanghai Cancer Center, Shanghai, China.

### Statistical Analysis

Association of age (young and elderly) with clinicopathological parameters was analyzed by chi-square (χ2) test. Continuous variables were analyzed using Student’s t-test. Survival curves were generated using Kaplan-Meier estimates, differences between the curves were analyzed by log-rank test. Multivariable Cox regression models were built for analysis of risk factors for survival outcomes. All statistical analyses were performed using the statistical software package SPSS for Windows, version 17 (Chicago: SPSS Inc, USA). Results were considered statistically significant when a two-tailed test of P<0.05 achieved.

## Results

### Patient Characteristics

We identified 69,835 eligible patients with CRC in SEER database during the 15-year study period (between 1988 and 2003). There were 37,130 (53.17%) males and 32,705(46.83%) females. The median ages in young and elderly groups were 36(15–40) and 64(41–74), respectively. The median follow-up period was 97(1–275) months. Patient demographics and pathological features are summarized in Table 1.

### Clinicopathological Differences between the Two Groups

When compared to elderly patients, in group of young ones, it was investigated that significant differences were found among the years of diagnosis (more frequent in recent years(2000–2003), P<0.001), race (less frequent in Caucasian race, p<0.001), pathological grading (more poor or undifferentiation in grade, p<0.001), histological type (more mucinous or signet-ring cancer, p<0.001), AJCC stage (more stage III, p<0.001), No. of LNs dissected (more cases with ≥12 LNs dissected, p<0.001) and LNR(more rN1, p<0.001). As regard to sex (p = 0.62) and primary site (p = 0.43), no significant differences between two groups were found. (Table 1).

**Table 1:** Characteristics of Patients from SEER Database by age.

	Total	Young Group	Elderly Group	P value
Characteristic	(n = 69835)	(n = 3014)	(n = 66821)	
Media foullow up(mo)		112	98	P<0.001
(IQR)		58–158	42–135	
Years of diagnosis				P<0.001
1988–1993	16017	609	15408	
1994–1999	21295	955	20340	
2000–2003	32523	1450	31073	
Sex				0.615
male	37130	1589	35541	
female	32705	1425	31280	
Race				P<0.001
Caucasian	55824	2183	53641	
African American	7390	431	6954	
Others	6443	391	6052	
Unknowns	178	9	169	
Primary site				0.434
Colon cancer	51372	2236	49136	
Rectal cancer	18463	778	17685	
Pathological grading				P<0.001
High/Moderate	54812	2167	52645	
Poor/undifferentiation	12644	723	11921	
Histological Type				P<0.001
Adenocarcinoma	61384	2455	58929	
Mucinous cancer	7804	474	7330	
Signet-ring cancer	647	85	562	
AJCC stage				P<0.001
I	4563	133	4430	
II	35867	1384	34483	
III	29405	1497	27908	
No. of LNs dissected				P<0.001
<12	37653	1037	36616	
≥12	32182	1977	30205	
Metastasis LNR				P<0.001
rN0(0)	40546	1521	39025	
rN1(0.01–0.20)	12814	722	12092	
rN2(0.21–0.60)	11482	541	10941	
rN3(>0.60)	4993	230	4763	

### Impact of Age on CRC Survival Outcomes

**Figure 1:**
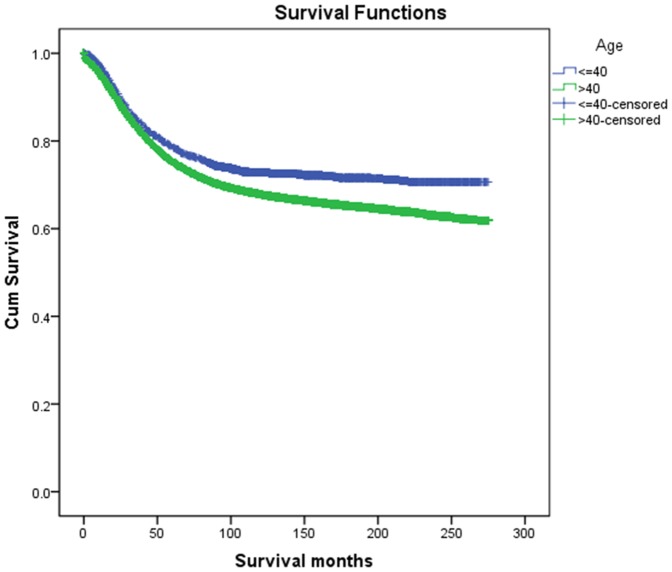
Survival curves in CRC patients according to age status. Young group vs. Elderly group, *χ2 = 35.84, P<0.001*.

The overall 5-year CCSS was 78.6% in young group and 75.3% in elderly group, which had significant difference in univariate log-rank test (P<0.001) (Fig. 1). Besides, early year of diagnosis (P<0.001), male (P<0.001), African race (P<0.001), rectal cancer (P<0.001), poor or undifferentiation tumor grade (P<0.001), mucinous or signet-ring cancer (P<0.001), higher AJCC stage(P<0.001), less number in LNs dissection(p<0.01) and higher metastatic LNR(P<0.001), were identified as significant risk factors for poor survival on univariate analysis(Table 2). When multivariate analysis with Cox regression was performed, we convinced all these factors as independent prognostic factors (Table 3). These included age (elderly, hazard ratio (HR) 1.42, 95% confidence interval (CI) 1.32–1.52), year of diagnosis (1994–1999, HR 0.83, 95% CI 0.80–0.86; 2000–2003, HR 0.74, 95% CI 0.71–0.76), gender (female, HR 0.87, 95%CI 0.85–0.90), race(African American, HR 1.19,95%CI 1.14–1.25;others, HR 1.59,95%CI 1.50–1.69), primary site(rectal cancer, HR 1.10,95%CI 1.07–1.14), pathological grading(poor or undifferentiation tumor, HR 1.32,95%CI 1.28–1.37), histological type(mucinous cancer, HR 1.10,95%CI 1.03–1.12; signet-ring cancer, HR 1.72,95%CI 1.54–1.92), AJCC stage(stage II, HR 2.91,95%CI 2.59–3.27; stage III, HR 4.83,95%CI 3.32–7.03), metastatic LNR(rN2, HR 1.92,95%CI 1.34–2.75; rN3, HR 3.23,95%CI 2.26–4.63), while the risk between rN0 and rN1 was not statistical difference(P = 0.45).

**Table 2:** Univariate survival analyses of CRC patients according to various clinicopathological variables.

Variable	n	5-year CCSS (%)	Log rank χ^2^ test		P
Years of diagnosis			333.65	P<0.001	
1988–1993	16017	70.9%			
1994–1999	21295	74.6%			
2000–2003	32523	78.3%			
Sex			105.86	P<0.001	
male	37130	74.4%			
female	32705	76.7%			
Age			35.84	P<0.001	
≤40	3014	78.6%			
41–74	66821	75.3%			
Race			195.16	P<0.001	
Caucasian	55824	76.0%			
African American	7390	69.3%			
Others*	6621	78.1%			
Primary site			144.24	P<0.001	
Colon cancer	51372	76.1%			
Rectal cancer	18463	73.6%			
Pathological grading			1128.17	P<0.001	
High/Moderate	54812	78.3%			
Poor/undifferentiation	12644	63.5%			
Histological Type			584.49	P<0.001	
Adenocarcinoma	61384	76.3%			
Mucinous cancer	7804	71.7%			
Signet-ring cancer	647	42.6%			
AJCC stage			7353.52	P<0.001	
I	4563	96.2%			
II	35867	85.4%			
III	29405	60.1%			
No. of LNs dissected			36.93	P<0.001	
<12	37653	74.7%			
≥12	32182	76.3%			
Metastasis LNR			11590.57	P<0.001	
rN0(0)	40546	86.6%			
rN1(0.01–0.20)	12814	72.3%			
rN2(0.21–0.60)	11482	56.3%			
rN3(>0.60)	4993	36.8%			

*including other (American Indian/AK Native, Asian/Pacific Islander) and unknowns.

**Table 3:** Multivariate Cox model analyses of prognostic factors of CRC.

Variable	Hazard Ratio	95%CI	P
Years of diagnosis			P<0.001
1988–1993	1.000		
1994–1999	0.827	0.798–0.857	
2000–2003	0.735	0.710–0.760	
Sex			P<0.001
male	1.000		
female	0.874	0.850–0.899	
Age			P<0.001
≤40	1.000		
41–74	1.415	1.315–1.523	
Race			P<0.001
Caucasian	1.000		
African American	1.194	1.136–1.254	
Others*	1.593	1.498–1.693	
Primary site			P<0.001
Colon cancer	1.000		
Rectal cancer	1.104	1.071–1.138	
Pathological grading			P<0.001
High/Moderate	1.000		
Poor/undifferentiation	1.322	1.279–1.366	
Histological Type			P<0.001
Adenocarcinoma	1.000		
Mucinous cancer	1.075	1.029–1.123	
Signet-ring cancer	1.716	1.537–1.917	
AJCC stage			P<0.001
I	1.000		
II	2.905	2.586–3.265	
III	4.826	3.315–7.026	
No. of LNs dissected			0.063
<12	1.000		
≥12	0.973	0.946–1.001	
Metastasis LNR			P<0.001
rN0(0)	1.000		
rN1(0.01–0.20)	1.149	0.802–1.646	
rN2(0.21–0.60)	1.918	1.340–2.746	
rN3(>0.60)	3.233	2.257–4.633	

*including other (American Indian/AK Native, Asian/Pacific Islander) and unknowns.

### Stratified Analysis of Age on Cancer Survival Based on Different Stages

We then made further analysis of age on 5-year CCSS in each stage. The results showed that young patients were significantly associated with better 5-year CCSS than elderly patients in univariate analysis in both stage II and III(P<0.001), but not in stage I (P = 0.605)(Table 4). And age was also validated as independent survival factor in multivariate Cox regression in stage II (elderly, HR 1.71, 95%CI 1.47–2.00, p<0.001) and stage III (elderly, HR 1.33, 95%CI 1.22–1.45, p<0.001) patients(Table 5).

**Table 4:** Univariate analysis of Age on CCSS based on different stages.

Variable	n	5-year survival (%)	Log rank χ^2^ test	P
**Stage I**				
Age			0.575	0.448
≤40	133	96.9%		
41–74	4430	96.2%		
**Stage II**				
Age			56.979	P<0.001
≤40	1384	90.5%		
41–74	24483	85.2%		
**Stage III**				
Age			29.677	P<0.001
≤40	1497	65.9%		
41–74	27908	59.8%		

**Table 5:** Multivariate Cox model analyses of prognostic factors of CRC on different stages.

Variable	Hazard Ratio	95%CI	P
**Stage II**			
Age			P<0.001
≤40	1.000		
41–74	1.709	1.465–1.995	
**Stage III**			
Age			P<0.001
≤40	1.000		
41–74	1.329	1.222–1.445	

P values refer to comparison between two groups and were adjusted for years of diagnosis, sex, age, race, primary site, pathological grading, tumor histotype, No. of LNs dissected, Metastasis LNR as covariates.

## Discussion

The current definition of young CRC patients remains controversial. Some studies used the cutoff age of 50 years [15], , while others used 30 years [14], [18] or 45 years [19]. But to date, majority of studies in the literature used the cutoff age of 40 years to denote a young patients with CRC [6], [12], [18], [20], [21]. This lack of a standard definition makes it difficult to make meaningful comparisons between different studies. We defined young patients using an upper limit of 40 years as most studies reported. In our study, the proportion of young patients with CRC with treatment of surgery has raised from 3.80%(609/15408) in year of 1988–1993 to 4.46%(1450/32523) in year of 1999–2003, which was consistent with the published epidemiologic feature [5], [22].

Various studies have reported poorer prognosis among young patients with CRC. Taylor et al. [21] in their review demonstrated that when compared to elderly patients, young patients less than 40 years of age presented with more advanced lesions and had lower survival rates. Similar results were reported by Marble et al [23] and Cusack et al [24]. This reduction in survival has been attributed to more advanced disease at diagnosis [6], [17], [20]. Tumor stage was very powerful factor effect long time survival rate. Poor tumor differentiation and mucinous and signet-ring cancer were also characteristic histological features in these patients [6]. It is well known that mucinous, signet-ring and poorly differentiated tumors tend to have a poorer prognosis compared to well and moderately differentiated tumors [25], Our study show that 5-year CCSS of adenocarcinoma, mucinous and signet-ring cancer was 76.3%, 71.7% and 42.6% respectively, signet-ring cancer is at extremely low rate.

In this cohort, despite the significantly higher incidence of poor prognostic factors such as poorly differentiated tumors, mucinous and signet-ring cancer, advanced AJCC stage in young group compared with the patients over 40 years of age, young CRC patients had a better 5-year CCSS, especially in stage II and stage III. This is demonstrated on both univariate and multivariate analysis. Our result is consist with some recently published articles [12], [13]. In this study, we included 3,014 young CRC patients, which is to date the largest number, and excluded CRC patients over 75 years old for short life expectation, which made our results more convincing. Young patients have a poorer biological behavior of carcinoma, but this is compensated by the better overall condition, faster postoperative recovery. In general, a good performance status is essential for the success of chemotherapy [26] and extensive lymphadenectomy. Clinicians are more inclined to gain all therapeutical options in young patients as they are at a better health condition and are more likely to tolerate toxicities associated with chemotherapy [27] while elderly patients always undertreated because of their age [28]. Adjuvant chemotherapy isn’t indication for stage I patients, which could help explaining why there weren’t significant difference in CCSS between young and elderly patients in this stage. Young patients also have a higher proportion of tumors demonstrating microsatellite instability, which are associated with a better prognosis [29]. Examination of at least 12 lymph nodes in the staging of CRC was recommended by the National Comprehensive Cancer Network (NCCN) and American Society for Clinical Oncology (ASCO) and about 65.59%(1977/3014) young CRC patients met this criterion compared with 45.20%(30205/66821) in elderly patients, which difference is statistical. Evaluation of an increasing number of lymph nodes has been shown to be associated with improved survival after resection of colon cancer [30], [31], but the level of significance just failed in multivariable Cox regression models in our study (P = 0.063). we also verified metastatic LNR as an independent prognosis factors by rN classification used by Zhang et al [32].

Although this is a large population-based study evaluating prognosis of young patients with CRC, it has several potential limitations. First, the SEER database lacks several important tumor characteristics (eg, perineural invasion and lymphovascular invasion), cancer therapy (neoadjuvant and adjuvant, quality of surgery). Thus, our analyses could not adjust for these potential confounding factors. Second, this data include only patients who had undergone surgical resection for CRC. As such, this group of patients can not represent CRC patients who had irresectable tumors or refused surgical intervention for various reasons. Still, our study has its convincing power regarding young CRC good survival rate after surgery.

In summary, compared to elderly patients, young patients with CRC aged 40 and below appear to have unique characteristics and have a higher CCSS after surgery although they presented with higher proportions of unfavorable biological behavior as well as advanced stage disease.
